# The association between long working hours and the metabolic syndrome: evidences from the 5th Korean National Health and Nutrition Examination Survey of 2010 and 2012

**DOI:** 10.1186/s40557-014-0053-9

**Published:** 2014-12-21

**Authors:** Jae Uk Jeong, Man Joong Jeon, Joon Sakong

**Affiliations:** Department of Occupational and Environmental Medicine, Yeungnam University Hospital, 170, Hyeonchung-ro, Nam-gu, Daegu, 705-802 Republic of Korea; Department of Preventive Medicine and Public Health, Yeungnam University College of Medicine, 170, Hyeonchung-ro, Nam-gu, Daegu, 705-802 Republic of Korea

**Keywords:** Long working hours, Metabolic syndrome, Central obesity

## Abstract

**Objectives:**

This study was conducted in order to evaluate the association between the working hours of Korean employees and the metabolic syndrome and the effects of long working hours on metabolic syndrome based on the 5th Korean National Health and Nutrition Examination Survey (2010-2012).

**Methods:**

Based on the 5th Korean National Health and Nutrition Examination Survey (2010-2012), 4,456 Korean employees without shift work, aged over 15, who work 30 hours or more per week were targeted in this study. The association between the general characteristics, including age, smoking, alcohol drinking, exercise, and the metabolic syndrome criteria defined by International Diabetes Federation (IDF) and weekly working hours were analyzed. In addition, the association between weekly working hours and the metabolic syndrome of the subjects stratified by gender was analyzed through multiple logistic regression analyses and generalized linear mixed model after adjusting the general characteristics.

**Results:**

In the results of stratified analysis by gender, in male subjects, in comparison with the 30-39 weekly working hours group, there were no significant adjusted odds ratios to the other working hours groups. In female subjects, in comparison with the 30-39 weekly working hours group, there were no significant adjusted odds ratios to the other working hours groups. In addition, no trend associations were observed among weekly working hour groups in both stratified genders.

**Conclusion:**

No significant differences in prevalence of metabolic syndrome of the subjects stratified by gender were found according to weekly increasing working hours. However, due to some limitations of this study, further prospective studies may be necessary for verification.

## Introduction

The effects of long working hours on health have been studied in the occupational and environmental medicine and various health care fields. However, there is considerable international variation in attitudes and practices relating to working hours, even within the developed world [1]. To protect workers’ health through limitations on working hours, International Labor Office (ILO) declared the Hours of Work (industry) Convention, 1919, and the Hours of Work (Commerce and Offices), 1930, which both stress the limits of the 8-hour work day and the 48-hour work week [2].

It is well established that cardiovascular disease is associated with the long working hours. Uehata et al. reported that the long working hours was a major cause of stress-associated cardiovascular diseases in middle-aged Japanese workers [3]. In a prospective cohort study conducted in UK, it was suggested that extended working hours was an independent factor that increased the risk of ischemic heart diseases [4]. Kang et al. reported that long working hours could increase the risk of coronary artery diseases [5].

In South Korea, yearly working hours are 2,163 hours, the second longest after Mexico among OECD countries [6]. Concern for Korean workers’ health status and quality of life is increasing. Recent domestic studies on long working hours include a case–control study which revealed the relationship between long working hours and cardiovascular disease [7]; a retrospective study which suggested that long working hours heightened the risk of work-related cerebro-cardiovascular disease [8]; a cross-sectional study conducted for male manual workers which showed that more than 60 working hours per week increased the risk of obesity [9].

It has been generally accepted that obesity and the metabolic syndrome are the major risk factors of cardiovascular disease. Obesity is known as an independent risk factor of cardiovascular disease [10], and a meta-analysis showed that people with metabolic syndrome are at increased risk of cardiovascular events [11].

As described in previous studies, long working hours have negative effects on health with a significant association with cardiovascular diseases. However, among studies on the negative effects of long working hours on health, only a few studies on the association between metabolic syndrome and long work hours have been conducted: Kobayashi et al. on the association between Japanese male workers’ weekly working hours and metabolic syndrome [12]. No study on the effects of long working hours on the metabolic syndrome in Korea has been reported.

Accordingly, in this study, we evaluate the association between long working hours of Korean employees and metabolic syndrome and the effects of long working hours on metabolic syndrome based on the 5th Korean National Health and Nutrition Examination Survey (2010–2012).

## Materials and methods

### Study subjects

The Korean National Health and Nutrition Examination Survey has been conducted in Korea to produce statistics that are necessary for understanding people’s health and nutrition statuses, and for establishing and evaluating health policies. In the 5th Korean National Health and Nutrition Examination Survey, rolling survey sampling for the years of 2010–2012 was used to establish dependent and homogeneous properties among the samples. Of the 25,534 persons who participated in the 5th Korean National Health and Nutrition Examination Survey (2010–2012), our study targeted subjects as full-time employees without shift work, aged over 15, except for self-employed workers and unpaid family workers. Because the data of 2012 exclude the questionnaire item asking the subject whether he or she works full-time or part-time, we regarded full-time work as 30 working hours per week. As a result, 4,456 subjects aged over 15, with 30 working hours or more per week, who responded to the weekly working hour items and the other questionnaire items and confirmed for the full criteria of the metabolic syndrome were selected for this study.

### Methods

Frequency analysis was performed in order to understand the demographic features of the subjects. In addition to working hours, items of smoking, alcohol drinking, and exercise, and the medical history of type 2 diabetes, hypertension, and dyslipidemia were also included, which could affect the prevalence of metabolic syndrome. Any item with a missing value was excluded from this study. Alcohol drinking habits were categorized according to two variables based on the Alcohol Use Disorder Identification Test (AUDIT) score (a group whose score is 7 or less, and the other 8 or more). Smoking was categorized according to nonsmoker and current smoker. Exercise was classified as who exercises (responding to any affirmative answer in exercise related questionnaires) and who does not.

There are several definitions of the metabolic syndrome, including National Cholesterol Education Program – Adult Treatment Panel III and International Diabetes Federation (IDF). The IDF definition was selected in this study to judge the criteria of metabolic syndrome because it reflects obesity more by setting it as the mandatory factor. Furthermore, some studies demonstrated that the IDF definition performed better in detecting the association between metabolic syndrome and cardiovascular disease [13]. The diagnostic criteria of metabolic syndrome of the IDF definition were: when central obesity (waist circumference ≥90 cm in males, ≥80 cm in females; or body mass index >30 kg/m^2^ in any cases) plus any two of raised triglyceride level (≥150 mg/dL or specific treatment for this lipid abnormality), reduced HDL cholesterol level (<40 mg/dL in males, <50 mg/dL in females or specific treatment for this lipid abnormality), raised blood pressure (systolic blood pressure ≥130 mmHg or diastolic blood pressure ≥85 mmHg or treatment of previously diagnosed hypertension), and raised fasting plasma glucose (≥100 mg/dL or previously diagnosed type 2 diabetes) were observed, the subject was assumed to have had metabolic syndrome. The weekly working hours were classified as 30–39, 40–48, 49–59, and 60 hours or more according to the Labor Standards Act, which defined regular work hours as 40 hours per week and the recommendation of ILO [2].

Pearson’s chi-square test, chi-square test for trend, and Fisher’s exact test were performed for analysis of the association among the weekly working hours, general characteristics, and metabolic syndrome criteria. One-way ANOVA and its linear trend were also performed for analysis of the association between the weekly working hours and the age of subjects. Multiple logistic regression analysis was performed using the metabolic syndrome prevalence as a dependent variable, and the general characteristics of the subjects (gender, age, smoking, alcohol drinking, and exercise), the weekly working hours as independent variables for calculation of the adjusted odds ratio and its 95% confidence interval (CI) adjusting for each dependent variable according to weekly working hours stratified by the subject’s characteristics. Trend association between the weekly working hours and metabolic syndrome in each stratified variables was assessed by generalized linear mixed model. Statistical significance was accepted when p-value was less than 0.05. SPSS WIN 21.0 was used for statistical analyses.

## Results

### General characteristics of the subjects

A total of 4,456 subjects (2,475 male subjects, 1,981 female subjects, respectively) participated in this study. Their mean age was 41.8 ± 12.3 years old (42.3 ± 11.7 years old in male, 41.3 ± 13.0 years old in female, respectively). Among male subjects, there were 239 (9.7%), 1,180 (47.7%), 614 (24.8%), and 442 (17.9%) individuals in the 30–39, 40–48, 49–59, and 60 or more weekly working hours groups, respectively. Among female subjects, there were 423 (21.4%), 1,025 (51.7%), 337 (17.0%), and 196 (9.9%) individuals in the 30–39, 40–48, 49–59, and 60 or more weekly working hours groups, respectively. The number of male subjects was significantly lower than that of female subjects in the 30–39 weekly working hours group, while it was significantly higher in the 40–48, 50–59, and 60 or more weekly working hours groups (p < 0.001).

Male subjects in the 40–48, 49–59, and 60 or more weekly working hours groups were significantly younger than those in the 30–39 weekly working hours group (p < 0.001). Female subjects in the 40–48 and 49–59 weekly working hours groups were significantly younger than those in the other weekly working hours groups (p < 0.001). The lowest proportion of current smoking in male subjects was observed in the 40–48 weekly working hours group, while the lowest proportion of current smoking in female subjects was observed in the 30–39 weekly working hours group (p < 0.05). There were no significant differences in the proportion of subjects in problem alcoholic drinking and exercise among weekly working hours groups of both genders.

Concerning past medical history, the highest number of cases of type 2 diabetes was observed in the 30–39 weekly working hours group of both genders. In cases of hypertension, the highest proportion of male subjects was observed in the 30–39 weekly working hours group and in the 60 or more weekly working hours group in female subjects. However, there were no significant differences in the proportion of subjects with dyslipidemia among weekly working hours groups of both genders (Table 1).

**Table 1:** **General characteristics of the subjects (N (%))**

**Characteristics**	**Male (n = 2475)**						**Female (n = 1981)**					
	**N (%)**	**Working hours per week**				**p**	**N (%)**	**Working hours per week**				**p**
		**30-39 (n = 239)**	**40-48 (n = 1180)**	**49-59 (n = 614)**	**≥60 (n = 442)**			**30-39 (n = 423)**	**40-48 (n = 1025)**	**49-59 (n = 337)**	**≥60 (n = 196)**	
Age (years)												
16-29	327 (13.2)	30 (12.6)	142 (12.0)	85 (13.8)	70 (15.8)	<0.001^†^	468 (23.6)	88 (20.8)	234 (22.8)	101 (30.0)	45 (23.0)	<0.001^†^
30-39	799 (32.3)	54 (22.6)	370 (31.4)	231 (37.6)	144 (32.6)		476 (24.0)	82 (19.4)	302 (29.5)	67 (19.9)	25 (12.8)	
40-49	686 (27.7)	48 (20.1)	344 (29.2)	168 (27.4)	126 (28.5)		487 (24.6)	103 (24.3)	263 (25.7)	77 (22.8)	44 (22.4)	
50-59	445 (18.0)	57 (23.8)	217 (18.4)	102 (16.6)	69 (15.6)		359 (18.1)	86 (20.3)	157 (15.3)	58 (17.2)	58 (29.6)	
≥60	218 (8.8)	50 (20.9)	107 (9.1)	28 (4.6)	33 (7.5)		191 (9.6)	64 (15.1)	69 (6.7)	34 (10.1)	24 (12.2)	
Mean (SD)	42.3 (11.7)	46.3 (14.4)	42.6 (11.4)	40.8 (10.5)	41.2 (11.8)	<0.001^‡^	41.3 (13.0)	43.5 (14.3)	40.1 (11.8)	40.4 (13.6)	44.5 (13.5)	<0.001^‡^
Smoking												
Yes	1135 (45.9)	115 (48.1)	501 (42.5)	300 (48.9)	219 (49.5)	0.014^†^	139 (7.0)	20 (4.7)	63 (6.1)	39 (11.6)	17 (8.7)	0.001^†^
No	1340 (54.1)	124 (51.9)	679 (57.5)	314 (51.1)	223 (50.5)		1842 (93.0)	403 (95.3)	962 (93.9)	298 (88.4)	179 (91.3)	
Drinking^*^												
≤7	1119 (45.2)	117 (49.0)	543 (46.0)	255 (41.5)	204 (46.2)	0.159^†^	1642 (82.9)	367 (86.8)	845 (82.4)	269 (79.8)	161 (82.1)	0.074^†^
≥8	1356 (54.8)	122 (51.0)	637 (54.0)	359 (58.5)	238 (53.8)		339 (17.1)	56 (13.2)	180 (17.6)	68 (20.2)	35 (17.9)	
Exercise												
Yes	2266 (91.6)	215 (90.0)	1095 (92.8)	562 (91.5)	394 (89.1)	0.091^†^	1754 (88.5)	378 (89.4)	916 (89.4)	294 (87.2)	116 (84.7)	0.222^†^
No	209 (8.4)	24 (10.0)	85 (7.2)	52 (8.5)	48 (10.9)		227 (11.5)	45 (10.6)	109 (10.6)	43 (12.8)	30 (15.3)	
DM												
Yes	107 (4.3)	19 (7.9)	55 (4.7)	20 (3.3)	13 (2.9)	0.009^†^	47 (2.4)	18 (4.3)	19 (1.9)	5 (1.5)	5 (2.6)	0.044^§^
No	2368 (95.7)	220 (92.1)	1125 (95.3)	594 (96.7)	429 (97.1)		1934 (97.6)	405 (95.7)	1006 (98.1)	332 (98.5)	191 (97.4)	
Dyslipidemia												
Yes	112 (4.5)	14 (5.9)	61 (5.2)	25 (4.1)	12 (2.7)	0.124^†^	61 (3.1)	19 (4.5)	29 (2.8)	7 (2.1)	6 (3.1)	0.243^†^
No	2363 (95.5)	225 (94.1)	1119^*^ (94.8)	589 (95.9)	430 (97.3)		1920 (96.9)	404 (95.5)	996 (97.2)	330 (97.9)	190 (96.9)	
Hypertension												
Yes	284 (11.5)	41 (17.2)	141 (11.9)	62 (10.1)	40 (9.0)	0.009^†^	169 (8.5)	43 (10.2)	71 (6.9)	25 (7.4)	30 (15.3)	0.001^†^
No	2191 (88.5)	198 (82.8)	1039 (88.1)	552 (89.9)	402 (91.0)		1812 (91.5)	380 (89.8)	954 (93.1)	312 (92.6)	166 (84.7)	

SD, standard deviation; DM, diabetes mellitus; p, p-value. ^*^problem alcohol drinking by AUDIT score, ^†^calculated by chi-square test, ^‡^calculated by one-way ANOVA, ^§^calculated by Fisher’s exact test.

### The prevalence of each criterion for metabolic syndrome of the subjects according to the weekly working hours

There were 711 (16.0%) subjects who met the criteria of metabolic syndrome. Among the IDF criteria for diagnosis of metabolic syndrome, in the components of raised blood pressure and fasting plasma glucose of male subjects, the highest prevalence was observed in the 30–39 weekly working hours group, followed by the 40–48 weekly working hours group. The prevalence of metabolic syndrome in male subjects among the weekly working hours groups was the highest in the 40–48 weekly working hours group. Among each of the IDF criteria for diagnosis of metabolic syndrome, in the components of raised blood pressure of female subjects, the highest prevalence was observed in the 30–39 weekly working hours group, followed by the 60 or more weekly working hours group. However, there were no significant differences in the prevalence of metabolic syndrome in female subjects among weekly working hours groups (Table 2).

**Table 2:** **Prevalence of each criteria for metabolic syndrome of subjects according to weekly working hours (N (%))**

**Criteria**	**Male (n = 2475)**							**Female (n = 1981)**						
	**Mean (SD)**	**Working hours per week**				**P** **	**p-trend** ^**††**^	**Mean (SD)**	**Working hours per week**				**p** **	**p-trend** ^**††**^
		**30-39 (n = 239)**	**40-48 (n = 1180)**	**49-59 (n = 614)**	≥**60 (n = 442)**				**30-39 (n = 423)**	**40-48 (n = 1025)**	**49-59 (n = 337)**	≥**60 (n = 196)**		
CO*	WC 84.0 (8.9) BMI 24.2 (3.2)	52 (21.8)	298 (25.3)	136 (22.1)	99 (22.4)	0.347	0.410	WC 75.8 (9.4) BMI 22.8 (3.4)	141 (33.3)	294 (28.7)	98 (29.1)	67 (34.2)	0.191	0.925
TG^†^	154.7 (117.6)	88 (36.8)	470 (39.8)	211 (34.4)	150 (33.9)	0.054	0.039	98.6 (68.4)	72 (17.0)	142 (13.9)	46 (13.7)	20 (10.3)	0.144	0.033
HDL^‡^	46.3 (10.4)	70 (29.3)	392 (33.2)	203 (33.1)	140 (31.7)	0.653	0.866	53.4 (11.1)	175 (41.4)	437 (42.7)	147 (43.6)	81 (41.3)	0.916	0.827
BP^§^	SBP 119.3 (14.6)	115 (48.1)	469 (39.7)	243 (39.6)	154 (34.8)	0.010	0.004	SBP 112.7 (15.8)	102 (24.1)	190 (18.5)	73 (21.7)	54 (27.6)	0.010	0.366
	DBP 80.2 (10.6)							DBP 73.3 (9.4)						
FPG^∥^	97.7 (20.8)	84 (35.1)	362 (30.7)	165 (26.9)	108 (24.4)	0.008	0.001	92.2 (16.7)	78 (18.4)	147 (14.4)	42 (12.5)	32 (16.4)	0.105	0.198
MS^¶^		34 (14.2)	224 (19.0)	91 (14.8)	381 (13.8)	0.021	0.074		75 (17.7)	143 (14.0)	50 (14.8)	33 (16.8)	0.286	0.692

SD, standard deviation; p, p-value; CO, central obesity; WC, waist circumference (cm); BMI, body mass index (kg/m^2^); TG, triglyceride (mg/dL); HDL, high density lipoprotein (mg/dL); BP, blood pressure (mmHg); SBP, systolic blood pressure (mmHg), DBP, diastolic blood pressure (mmHg); FPG, fasting plasma glucose (mg/dL); MS, metabolic syndrome.

*central obesity (WC ≥90 cm or BMI >30 kg/m^2^ in male, WC ≥80 cm or BMI >30 kg/m^2^ in female), ^†^raised TG ≥150 mg/dL or being treated, ^‡^reduced HDL (≤40 mg/dL in male, ≤50 mg/dL in female or being treated), §raised BP (SBP ≥130 mmHg or DBP ≥85 mmHg or being treated), ∥raised FPG ≥100 mg/dL or previous diagnosed type 2 diabetes, ^¶^person with the metabolic syndrome must have central obesity plus any two of the four factors (FPG ≥100 mg/dL or previously diagnosed type 2 diabetes, TG ≥150 mg or being treated, reduced HDL (<40 mg/dL in male, <50 mg/dL in female) or being treated, and raised BP (SBP ≥130 mmHg or DBP ≥85 mmHg) or being treated.

**calculated by chi-square test, ^††^calculated by chi-square test for trend.

### Odds ratios for metabolic syndrome of the subjects according to the weekly working hours stratified by gender

We used age, whether or not smoking, problem alcoholic drinking, exercise as variables in multiple logistic regression analysis and calculated adjusted odds ratio and its 95% confidence interval by adjusting for each variables. In the results of stratified analysis by gender, in male subjects, in comparison with the 30–39 weekly working hours group, there were no significant adjusted odds ratios to the other working hour groups. In female subjects, in comparison with the 30–39 weekly working hours group, there were no significant adjusted odds ratios to the other working hour groups. Also, there were no trend associations among weekly working hours groups in both stratified genders (Table 3).

**Table 3:** **Odds ratios for metabolic syndrome of subjects according to weekly working hours stratified by gender (N =4456)**

**Weekly working hours**	**Male (n = 2475)**			**Female (n = 1981)**		
	**N**	**OR** * **(95% CI)**		**N**	**OR** * **(95% CI)**	
30-39	239	1.00	(Reference)	423	1.00	(Reference)
40-48	1180	1.48	(0.99-2.21)	1025	1.03	(0.74-1.45)
49-59	614	1.12	(0.72-1.73)	337	0.97	(0.63-1.48)
≥60	442	1.04	(0.66-1.65)	196	0.89	(0.55-1.44)
P for trend^†^		0.767			0.568	

OR, odds ratio; CI, confidence interval.

*adjusted for age, smoking, alcoholic drinking, and exercise.

^†^calculated by generalized linear mixed model.

## Discussion

Studies on metabolic syndrome have been actively conducted in recent decades due to its association with cardiovascular diseases. In 1923, Kylin understood hypertension, hyperglycemia, and gout as components of a syndrome. In 1988, Gerald Reaven introduced hypertension, hyperglycemia, hypertriglyceridemia, and low HDL as Syndrome X, which was a risk factor of cardiovascular diseases [14]. Since then, central obesity and insulin resistance have been understood as the core symptoms of metabolic syndrome. World Health Organization (WHO) suggested that the syndrome be called metabolic syndrome in 1998. Each metabolic syndrome factor is known to increase the prevalence of type 2 diabetes and cardiovascular diseases, and their mortality. When these factors are combined, the situation becomes much worse [11].

Findings of a few studies have indicated that long time shift-work is associated with metabolic syndrome. A study conducted for female workers in fabric plants showed positive association between shift-workers and metabolic syndrome in comparison with daytime workers [15]. A cross-sectional study conducted for female hospital nurses and male manual workers in Korea also showed an association of shift-work with metabolic syndrome [16]. Therefore, to exclude the effect of shift-work on metabolic syndrome, we targeted only workers without shift work as subjects. Self-employed workers and unpaid family workers were excluded from this study because there are differences in health behaviors and working conditions among self-employed workers, unpaid family workers, and wage workers.

The results of this study suggest that long working hours may not be associated with metabolic syndrome, with no significant odds difference in increasing weekly working hours. These insignificant results remained unchanged after adjustments for gender, age, smoking, alcohol drinking habit, and exercise. In stratified analysis by gender, no clear association was observed between long working hours and risk of metabolic syndrome in male group and in female group as well.

These findings are in accordance with those of a cross-sectional study of Japanese employees [17], showing a negative association between long working hours and the prevalence of diabetes. Another meta-analysis [18] also did not support the evidence that long working hours are directly associated with an increased risk of type 2 diabetes. A cross-sectional study on the association between daily working hours and metabolic syndrome in male Japanese workers reported significant outcome in subjects aged 40 or more, but, no significant association was observed between working hours and prevalence of metabolic syndrome when stratified by age [12]. However, a longitudinal study conducted for male workers in local public institutions of Japan suggested that long working hours increase the onset risk of hypertriglycemia [19]. In addition, a recent study conducted for Korean adults [5] demonstrated that long working hours are significantly related to risk of coronary heart disease. These discordant results among the studies are not clearly explained, but a plausible assumption might include the differences in the characteristics of each subjects and inconsistent categorization of working hours.

There are some possible explanations on the finding of our study. First, this study is based on the hypothesis that long working hours can be a potent negative health effect factor. But according to the socioeconomic status of subjects, for example, who are engaged in the specialized or professional job, long working hours can be voluntary and motivational or long working hours may not be hazardous factors of health. Therefore, there is suspicion that long working hours is not a unilateral negative health factor [20]. Second, so-called healthy worker selection effect may exert influence on the result of this study. That is, it is possible that subjects who have health problems cannot perform long hour working and vice versa, subjects who can handle long working hours without health problems survive. Future studies on long working hours and health effect are required in order to take it into account. Third, there is a possibility that metabolic syndrome may be a weaker predictor for detecting the risk of cardiovascular diseases than other predictors such as type 2 diabetes. Fourth, in our analysis, adjustments of some potent factors that could affect the prevalence of metabolic syndrome are missing such as dietary status and socioeconomic status of subjects.

To the extent of our knowledge, this is the first study investigating the association between long working hours and metabolic syndrome in Korea. And the IDF definition to evaluate metabolic syndrome in this study would be an advantage. Several criteria of metabolic syndrome have been reported by various institutions, and it has been an increasing argument as to which criteria are more appropriate as an effective predictor of cardiovascular disease. In some studies, the IDF criteria showed a fair level of agreement with WHO criteria [21,22]. Finally, this study includes population-based large scale data, which would be one of the strengths of our study.

However, this study is not without limitations. First, as the study is based on cross-sectional analysis, it is possible to make causal inferences from the findings of this study, even though it is unlikely that subjects who meet the criteria of metabolic syndrome tend to be work long hours. Second, this study did not distinguish between manual labor workers and white-collar workers. Many research studies have indicated the disparities in the prevalence of metabolic syndrome between manual workers and non-manual workers [23,24], but this consideration was not reflected in our study. Third, there is a possibility that whole life working duration would affect the results of this study, but data available in our study did not include items on history of working time of subjects. Fourth, the classification of working hour group is not based on strict evidence, and categorization of weekly working hours may cause loss of information. Fifth, because weekly working hours were assessed by self-reported questionnaire in this study, we cannot rule out informational error caused by misclassification.

## Conclusion

No significant differences in prevalence of metabolic syndrome were found according to increasing weekly working hours in this study. However, as described in previous studies, long working hours could negatively affect the health status, especially the cardiovascular system and its related metabolic factors. Additional prospective studies on the association between long working hours and metabolic syndrome may be necessary in the future.
